# MAIG-Net: Unsupervised Remote Sensing Road Extraction Combining Multi-Layer Adversarial Learning and Intermediate Domain Guidance

**DOI:** 10.3390/s26165197

**Published:** 2026-08-17

**Authors:** Chengqi Bao, Guangwu Chen, Wenbo Jin, Jingyu Yang, Shaoyuan Li

**Affiliations:** 1 School of Automation and Electrical Engineering, Lanzhou Jiaotong University, Lanzhou 730070, China; 11250696@stu.lzjtu.edu.cn (C.B.); cgwyjh1976@126.com (G.C.);; 2 Gansu Provincial Interdisciplinary Center for Air-Rail Intelligent Transportation and Energy Interconnection, Lanzhou 730070, China; 3 School of Electronic and Information Engineering, Lanzhou Jiaotong University, Lanzhou 730070, China; 11250736@stu.lzjtu.edu.cn

**Keywords:** road extraction, unsupervised domain adaptation, ground sampling distance, Fourier domain adaptation, intermediate domain guidance, adversarial learning

## Abstract

In unsupervised cross-domain remote sensing road extraction, severe data distribution shifts and heterogeneous background interference significantly constrain model performance. In addition to appearance shift, source and target images acquired by different sensors often present roads at unequal physical scales. To address this, MAIG-Net combines a target-label-free ground-sampling-distance (GSD) rule that matches the physical field of view of the two domains, an intermediate domain constructed by Fourier domain adaptation (FDA) that transfers only low-frequency target appearance onto labeled source images while preserving the complete source phase and road labels, and Domain-Invariant Feature Alignment (DIFA) modules that perform reliability-weighted, topology-conditioned adversarial alignment at three encoder depths. An exponential-moving-average (EMA) teacher supplies detached reliability and topology conditions for the domain discriminators; no target prediction is used as a direct segmentation label. Under a matched protocol with a fixed 30-epoch budget and three seeds, MAIG-Net improves the mean road IoU from 0.327 to 0.376 on SpaceNet→DeepGlobe and from 0.450 to 0.458 on SpaceNet→Massachusetts relative to source-only training, with consistent per-seed gains in both directions; the smaller Massachusetts effect is directionally reproduced on a previously untouched validation holdout. Reverse adaptation, reliability perturbations, computational cost, and failure cases are further reported to delimit the applicability of the method.

## 1. Introduction

**Figure 1:**
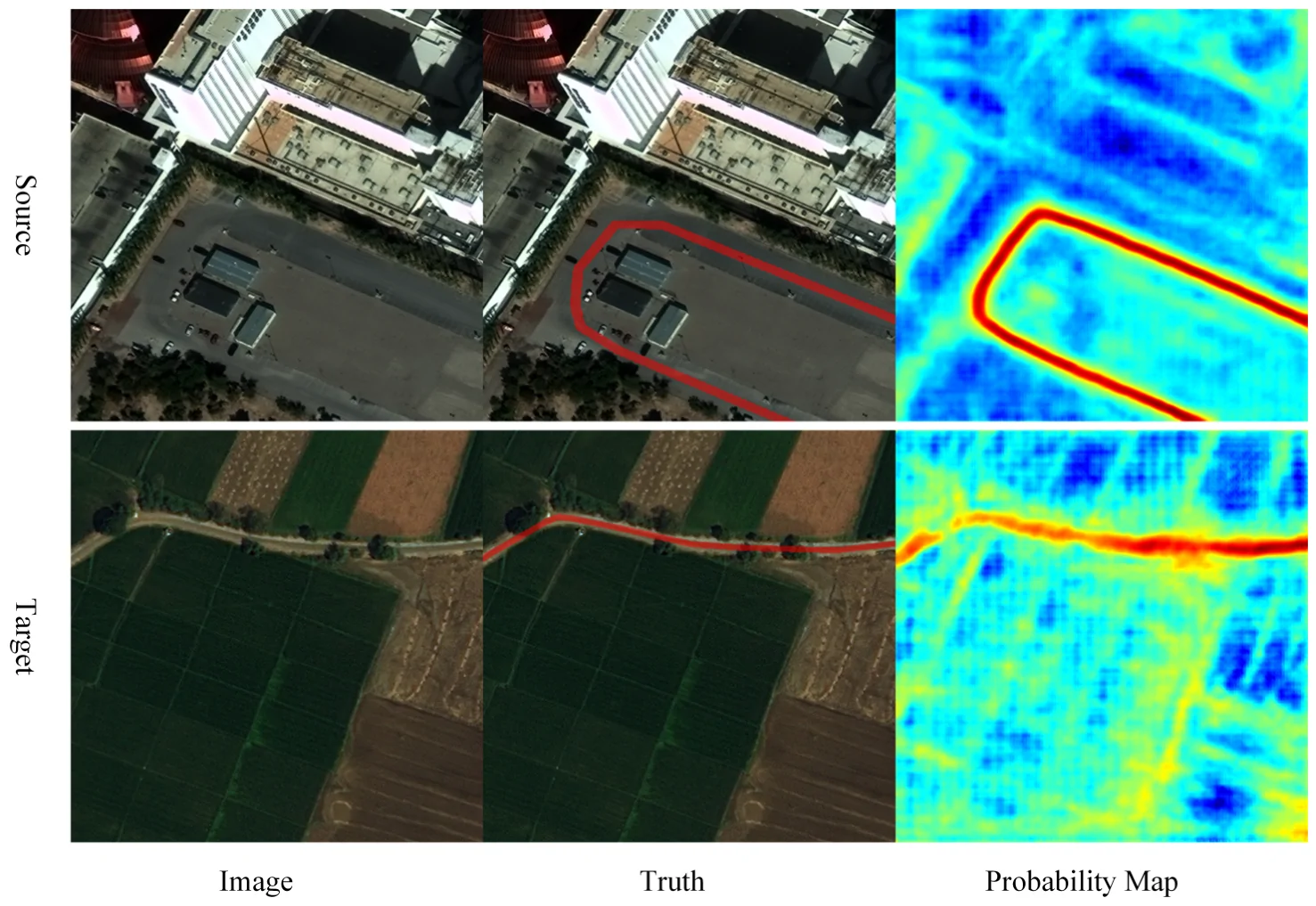
Comparison of extraction results across different domains.

Deep neural networks have demonstrated exceptional feature modeling capabilities in road extraction tasks under constrained environments. However, existing high-performance models often heavily rely on large-scale, high-quality annotated data. Furthermore, when facing cross-regional and cross-sensor application scenarios, models trained in the source domain often exhibit severe performance degradation in the target domain due to significant differences in imaging conditions, regional topography, and road materials, as shown in the second row of Figure 1. This domain shift phenomenon, caused by data distribution discrepancies, has become a core bottleneck restricting road extraction technology from transitioning from laboratory research to large-scale engineering implementation [1].

Unsupervised Domain Adaptation (UDA) aims to alleviate this problem by utilizing unlabeled target domain data. However, for the specific task of road extraction, existing UDA methods face unique challenges. First, the forced alignment of global feature distributions often leads to negative transfer. Mainstream adversarial learning methods tend to minimize the discrepancy between the overall distributions of the source and target domains. However, when confronting cross-scenario tasks with extreme differences, this global strategy easily destroys the local geometric features of elongated targets like roads, leading to blurred or broken edges. Second, the feature extraction of road targets is highly dependent on their surrounding background environments. In cross-domain transfer, although the core semantics of the roads themselves are similar, their co-existing background environments undergo drastic changes. Existing methods struggle to strip this background variance from the road features, rendering the model unable to correctly identify roads against the new backgrounds of the target domain. Finally, self-training strategies based on pseudo-labels are susceptible to noise accumulation. Although multi-stage self-training or mix-up augmentation strategies have proven effective in weakly supervised or intra-domain scenarios, in cross-domain scenarios, initial pseudo-labels often contain numerous errors. These errors are continuously amplified during the iterative process, causing the model’s adaptive capacity for hard samples to stagnate [2].

In the practical application of high-resolution remote sensing imagery, models trained on source domains frequently face severe domain shift problems in target domains due to significant differences in sensor imaging mechanisms, regional topography, and illumination conditions. To mitigate the performance degradation caused by data distribution discrepancies, unsupervised domain adaptation and semi-supervised learning technologies have been widely introduced into the field of remote sensing road extraction [3]. Existing research methods are mainly developed along the following technical trajectories, which can be categorized into three types.

The first category encompasses methods based on adversarial learning for global and local alignment. Utilizing Generative Adversarial Networks (GANs) or domain discriminators to achieve cross-domain distribution alignment is a classic approach to alleviating domain shift. Benjdira et al. [4] pioneered the demonstration of the effectiveness of adversarial training in reducing cross-domain visual distribution differences. However, pure global alignment easily disrupts the elongated geometric structure of roads. Lu et al. [5] explicitly pointed out the limitations of a single global alignment and proposed a cascaded adversarial framework combining local regional features. Chen et al. [6] introduced a multi-scale discriminator, effectively preserving the spatial structure of road networks during the adversarial process. To overcome severe physical deformations caused by cross-sensor variations, Iqbal et al. [7] specifically introduced structural constraints.

The second category is based on the concept of self-training with pseudo-labels and consistency constraints, utilizing high-confidence prediction results from the target domain for iterative self-training and fine-tuning. To enhance the reliability of pseudo-labels, Tarvainen et al. [8] constructed a Teacher–Student architecture via exponential moving average (EMA). Building upon this, recent studies have proposed more refined noise suppression strategies. Zhang et al. [9] progressively filtered noise by gradually increasing the confidence threshold; meanwhile, Gao et al. [10] and Li et al. [11] optimized the intra-class compactness of the feature space by introducing multi-view consistency and pixel-level contrastive learning. Although consistency constraints and staged strategies can improve robustness, the self-training paradigm is generally constrained by confirmation bias. Models are highly prone to continuously reinforcing their own erroneous predictions during iteration, and it is difficult to effectively mine and correct hard samples with lower confidence.

The final category consists of methods based on data mix-up augmentation and feature decoupling. To enrich the data representation of the target domain at the source and break through the domain shift bottleneck, research in recent years has begun to shift its focus toward cross-domain mixing and decoupling strategies. Song et al. [12] proposed a structure-aware mix-up augmentation strategy, simulating complex scenarios by dynamically swapping foreground regions. Subsequently, decoupling in the frequency domain was further explored; for example, Ma et al. [13] attempted to strip domain-invariant content from domain-specific styles. Lu et al. [14] in their latest research, explored an adaptive target disentanglement mechanism to strengthen the inter-class discriminative power of features. Existing domain adaptation methods have made progress in narrowing global distribution differences and mining unsupervised information of the targets. However, when confronted with the highly structured nature of roads and the extreme heterogeneity of their symbiotic backgrounds, most existing mix-up augmentation or disentanglement methods remain at the level of implicit global constraints.

Recent studies further explore source-free domain adaptation (SFDA), transformer-based UDA, and explicit content–style decomposition. SFDA methods [2] cannot access source data during target adaptation, which suits privacy- or storage-constrained scenarios but also precludes the use of labeled source road geometry at that stage; the present study belongs to standard UDA, which retains the labeled source domain. De-GLGAN [13] combines decomposition with global–local adversarial learning, but its released experiments cover Potsdam–Vaihingen and LoveDA rather than the road directions studied here. The adaptive disentangled target representation of Lu et al. [14] has no identifiable published result on the SpaceNet, DeepGlobe, or Massachusetts road directions. These methods motivate separating appearance from structure, whereas the present study additionally controls physical image scale before adaptation and conditions multi-level alignment on road topology.

Adjacent remote sensing and thin-structure studies further inform the architectural design of this work: localization–balance–affinity mechanisms for salient objects motivate boundary preservation of elongated structures [15]; context association for landslide extraction motivates combining local and contextual evidence [16]; detail–semantic collaboration in depth estimation motivates multi-depth representations [17]; and visual-attention-guided crack detection illustrates thin-structure reasoning under background clutter [18]. Because these studies address salient object detection, landslide mapping, depth estimation, and crack detection rather than road domain adaptation, they are cited only as architectural motivation, and no performance number is transferred across tasks.

The intermediate-domain strategy adopted in this paper is motivated by the same principle that underlies the mix-up methods discussed above, namely the insertion of a transitional distribution between the source and target domains so that a severe global shift is decomposed into a sequence of smaller, more tractable adaptation steps. Its realization, however, differs in an essential respect: the transition is constructed in the frequency domain rather than in the spatial domain. A natural spatial realization, exemplified by the ClassMix paradigm [19], composites source foreground regions onto target backgrounds. Two limitations of such spatial composites motivate the present design. First, cut-and-paste operations inevitably introduce artifacts along the mask boundaries. Second, and more fundamentally, supervising the composited result requires target pseudo-labels, thereby reintroducing the very noise-accumulation problem that the intermediate domain is intended to alleviate. MAIG-Net therefore constructs the transitional bridge in the frequency domain: the low-frequency appearance of the target domain is interpolated onto entire labeled source images, while the complete source phase and the associated road labels are preserved. Each intermediate sample consequently exhibits target-like appearance over precisely known source structure and remains fully supervised by source labels. The guiding role of the intermediate domain is thus preserved—it anchors the appearance path of the network and conditions the multi-level alignment—while the constraints associated with it operate entirely without target pseudo-labels (Section 2.4).

To delimit the contribution of this paper explicitly, the components of MAIG-Net are attributed at three levels. (1) Standard components: the EMA Teacher–Student mechanism [8], Fourier domain adaptation (FDA) frequency interpolation [20], and gradient-reversal adversarial learning [21]. (2) Module-level adaptations: spatial–frequency DIFA transforms and equal-width domain discriminators inserted after encoder stages 2, 3, and 4 of a road segmentation backbone. (3) Study-specific contributions: (i) a target-label-free ground-sampling-distance (GSD) rule that matches the physical fields of view of the source and target domains before resizing; (ii) a whole-image, source-supervised appearance path that alters only centered low-frequency amplitudes while preserving the complete source phase and road labels, serving as the intermediate domain; and (iii) guarded, reliability-weighted, topology-conditioned adversarial alignment at three encoder depths. MAIG-Net deliberately employs neither spatial ClassMix mixing [19], nor a prototype bridge, path consistency, a direct target pseudo-label segmentation loss, or a gradient penalty; the rationale for these design exclusions is given in Section 2.4 and Section 2.5.

## 2. Materials and Methods

To address the issues of inter-domain distribution gaps and feature confusion in road extraction, this paper proposes an unsupervised road extraction method based on multi-layer adversarial learning and intermediate domain guidance. First, the framework introduces adversarial learning at different feature levels of the backbone network, constructing a comprehensive alignment mechanism that ranges from shallow visual representations to deep semantics. To further bridge the significant inter-domain distribution gap, an intermediate domain guidance strategy is introduced. In MAIG-Net, the intermediate domain is constructed in the frequency domain: target low-frequency appearance is interpolated onto labeled source images while the complete source phase and road labels are preserved, so that the intermediate samples carry source structure under target-like appearance and remain fully supervised by source labels. This converts drastic global distribution transfer into a progressive appearance adaptation process that is easier for the network to learn. Building upon this, a dual constraint mechanism based on intermediate domain guidance is introduced: a pixel-level reliability constraint and a topology-consistency constraint jointly decide which detached teacher predictions may condition the domain discriminators. Throughout this section, $\left(x_{s},y_{s}\right)$ denotes a labeled source image and its road mask, and $x_{t}$ denotes an unlabeled target image; target masks are never used for training, checkpoint selection, threshold selection, or sampling geometry.

### 2.1. Overall Network Architecture

**Figure 2:**
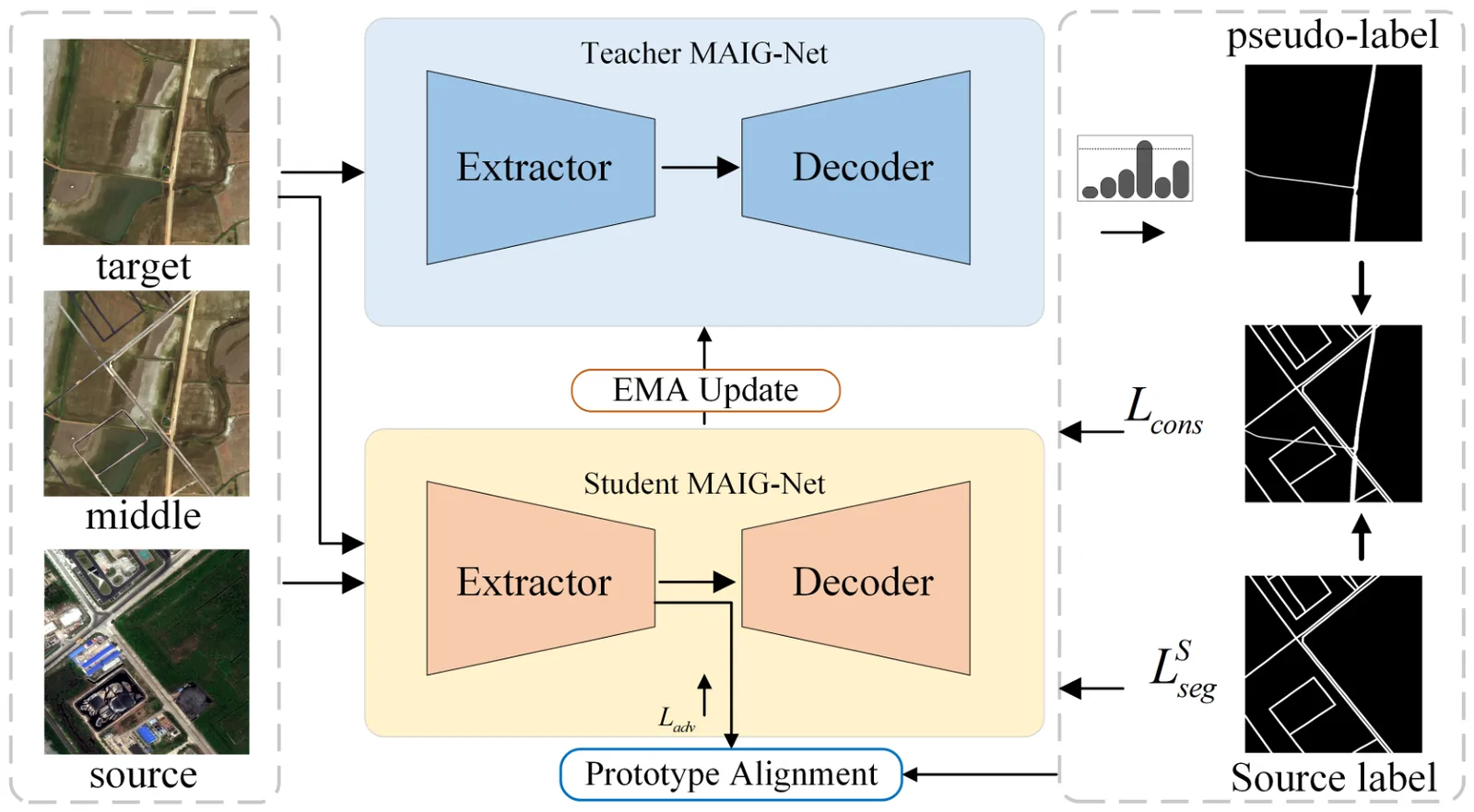
One training iteration of MAIG-Net. GSD-matched source and target crops enter the shared student; the FDA intermediate image is supervised by the source label; the detached EMA teacher supplies reliability and topology conditions for the training-only stage-2/3/4 discriminators. At inference, the EMA encoder–decoder and the DIFA transforms are retained, while the image-level FDA and all discriminators are removed.

The overall architecture of the proposed unsupervised road extraction method is illustrated in Figure 2. This framework adopts a Teacher–Student collaborative architecture as its foundation, with both feature extractors utilizing ResNet-34 as the backbone network. The student network receives the source domain, intermediate domain, and target domain inputs and performs gradient updates to learn cross-domain invariant features. The teacher network does not participate in backpropagation; instead, it periodically integrates the weights of the student network through an Exponential Moving Average (EMA) strategy. The parameter update formula is as follows:

(1) $$\theta_{T}^{\left(k\right)}=\alpha \;\theta_{T}^{\left(k-1\right)}+\left(1-\alpha \right)\;\theta_{S}^{\left(k\right)}$$

where $\theta_{T}^{\left(k\right)}$ and $\theta_{S}^{\left(k\right)}$ denote the parameters of the teacher and student networks after the *k*-th optimizer step, the superscript *k* indexes the optimizer steps (the update is applied once per step), and α∈[0,1) is the smoothing coefficient, fixed to α=0.999 (Section 3.4 compares alternatives). In MAIG-Net, the teacher provides only detached reliability and topology conditions for the domain discriminators; its target predictions are not used as segmentation labels or prototype targets.

GSD-aware sampling. Resizing native images from different sensors to the same pixel dimensions can present roads at different physical widths, which confounds appearance alignment with scale mismatch. For a network crop of P=512 pixels, we define a target-label-free physical field of view

(2) $$L=\min\left(384\;m,\;g_{t}P\right)$$

where $g_{t}$ is the nominal target-domain GSD in meters per pixel. A native image from domain *d* with GSD $g_{d}$ is cropped to approximately $\mathrm{round}(L/g_{d})$ pixels and resized to P×P. SpaceNet→DeepGlobe therefore uses a 256-m field of view (853 source pixels and 512 target pixels; effective GSD 0.50 m/pixel), while SpaceNet→Massachusetts uses 384 m (1280 source pixels and 384 target pixels; effective GSD 0.75 m/pixel). At evaluation, the complete target image and mask are resized to the effective GSD with spatial dimensions rounded to multiples of 32, using nearest-neighbor interpolation for masks. Equation (2) depends only on published dataset geometry and image dimensions and never reads a target label.

Intermediate domain construction. Given a GSD-matched source–target pair, the two images are transformed by a two-dimensional FFT after undoing ImageNet normalization. Let $A_{s}$ and $\phi_{s}$ denote the source amplitude and phase, and $A_{t}$ the target amplitude, each indexed by the two-dimensional frequency coordinate *u*. Inside a centered low-frequency window $\Omega_{\beta }$ with side ratio β=0.05, the amplitude is interpolated with strength λ∈[0,1] as

(3) $$A_{st}\left(u\right)=\left\{\begin{array}{cc} \left(1-\lambda \right)A_{s}\left(u\right)+\lambda A_{t}\left(u\right), & u\in \Omega_{\beta }\\ A_{s}\left(u\right), & u\notin \Omega_{\beta } \end{array}\right.$$

and the intermediate (styled source) image is reconstructed as

**Figure 3:**
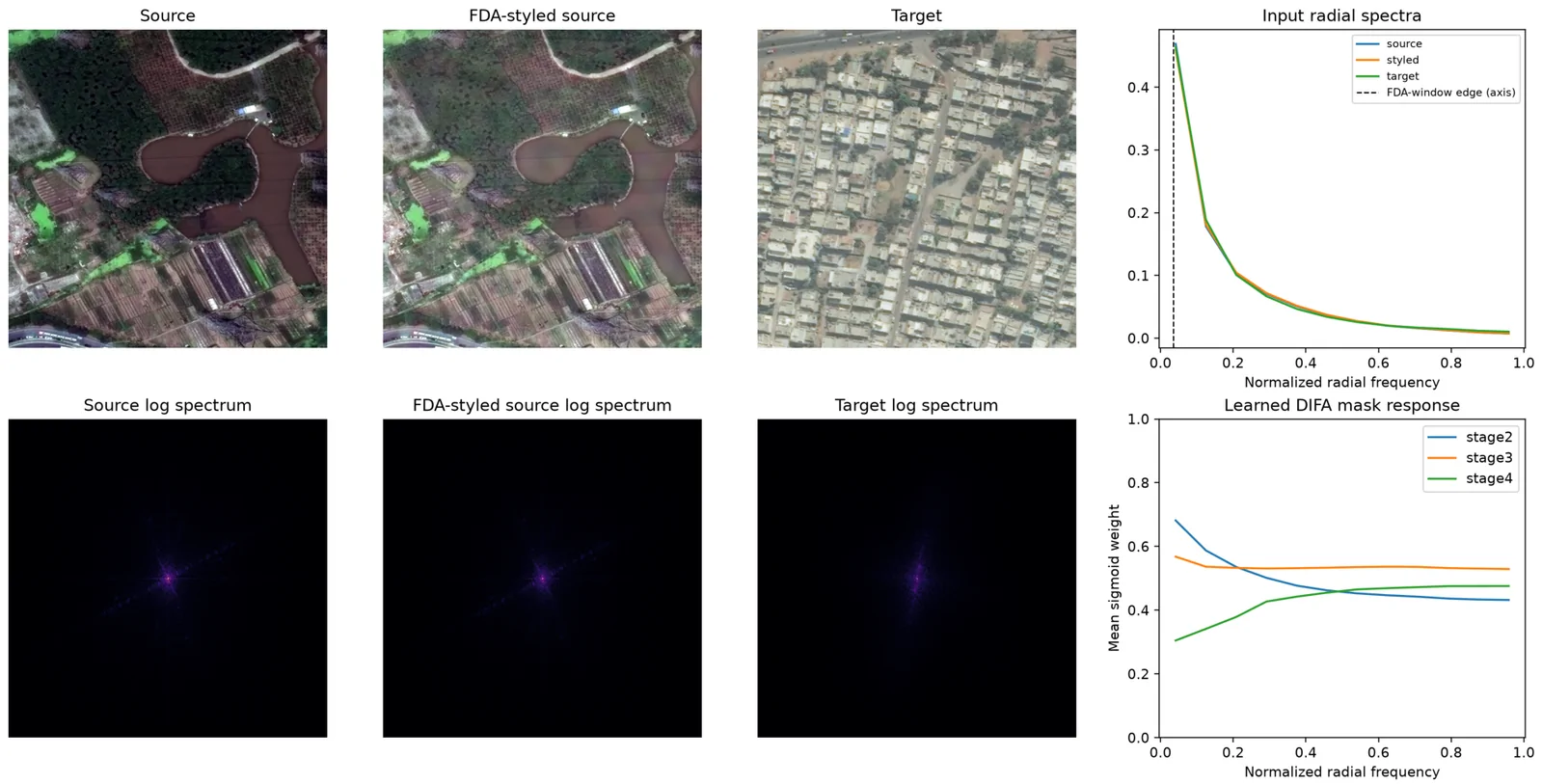
Target-label-free frequency analysis over 64 GSD-matched source–target pairs. The panels distinguish the training-only input-level FDA (source, styled source, and target images with their spectra) from the learned feature-level DIFA amplitude masks retained at inference (radial profiles and FFT shift-centered mean masks).

(4) xst=ReIFFT2Astejϕs.

where *j* denotes the imaginary unit and Re[·] takes the real part. Because the complete source phase and spatial support are retained, both $x_{s}$ and $x_{st}$ are supervised by the same source label $y_{s}$. After a 15-epoch source-only warm-up, the interpolation strength λ increases from 0.15 to 0.45 with uniform jitter of at most 0.05, clipped to this interval. Unlike spatial cut-and-paste constructions, this whole-image operation creates no mask boundary artifacts and requires no target pseudo-label. Figure 3 visualizes the input-level FDA operation and, separately, the learned feature-level responses described in Section 2.3.

### 2.2. MAIG-Net

To achieve high-precision cross-domain road extraction and effectively overcome both the significant variations in intrinsic road morphology and materials across different domains and the severe interference caused by heterogeneous backgrounds, MAIG-Net (Multi-layer Adversarial Intermediate Guidance Network) improves upon the traditional encoder–decoder architecture. The network primarily consists of two components: an encoder based on multi-layer adversarial learning and a decoder that restores the topological structure of the roads. The encoder employs ResNet-34 as its backbone, capturing multi-scale road semantic information through four feature extraction stages at different levels. To bridge the distribution gap between the source and target domains, Domain-Invariant Feature Alignment (DIFA) modules are embedded after encoder stages 2, 3, and 4, whose channel widths for ResNet-34 are 128, 256, and 512, respectively. Stage 1 remains part of the shared encoder and receives source segmentation gradients through the normal backbone path, but it is excluded from adversarial alignment because it carries the largest spatial maps and is dominated by domain-specific shallow edges and textures.

The decoder follows the D-LinkNet design: a cascaded dilated center block with dilation rates 1, 2, 4, and 8 enlarges the receptive field at the deepest stage, and skip-connected decoding blocks progressively restore the spatial resolution, ultimately outputting a road probability map at the input resolution. The DIFA outputs replace the corresponding unenhanced encoder features both in the skip connections and along the main path, and the entire DIFA path remains active at inference.

### 2.3. Domain-Invariant Feature Alignment

**Figure 4:**
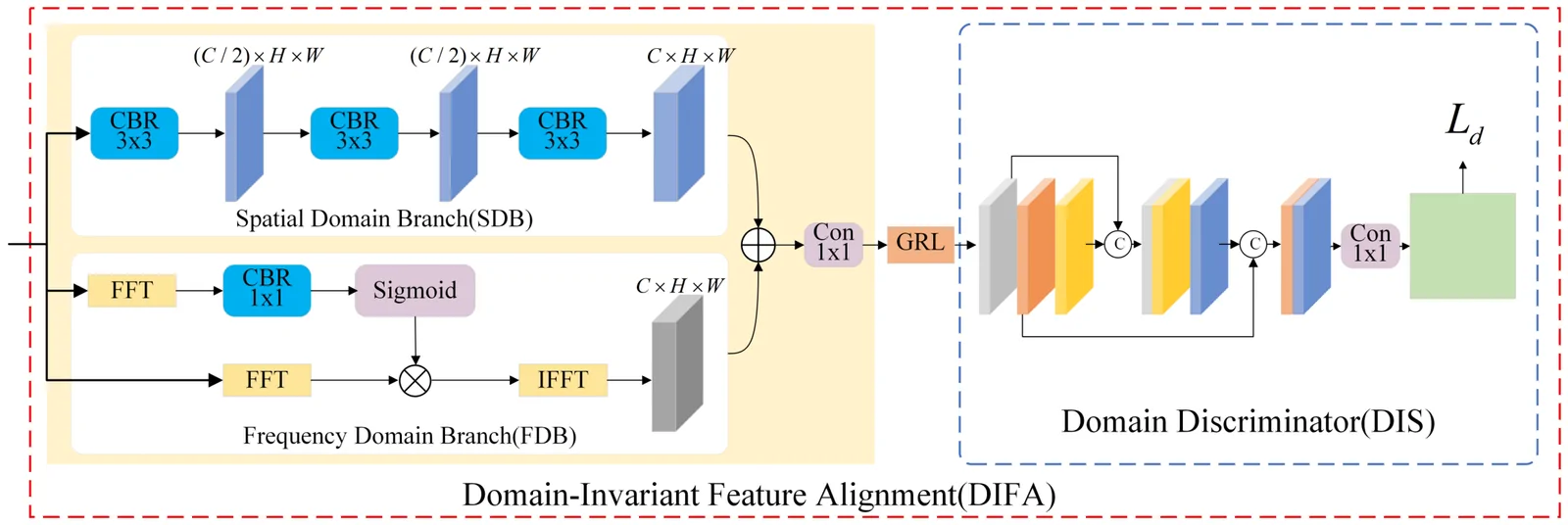
Schematic diagram of the DIFA module.

The Domain-Invariant Feature Alignment (DIFA) module is specifically designed in this paper. As illustrated in Figure 4, this module performs feature extraction through a dual-branch architecture consisting of the spatial and frequency domains. It achieves complementary advantages between local details and global topology, thereby realizing the mining and alignment of common road features across domains.

First is the Spatial Domain Branch (SDB), which focuses on capturing local textures and edge details of roads. The input feature maps sequentially pass through three groups of 3×3 CBR (Convolutional layer, Batch Normalization layer, ReLU activation function) processing units. Due to the limited local receptive field of standard convolutions, this branch is primarily responsible for the fine characterization of microscopic features. During this process, the branch employs a channel bottleneck strategy, where the number of feature channels is first reduced from *C* to C/2, and then restored to the original *C* channels through the final 3×3 convolution. This design of initial dimensionality reduction followed by expansion can effectively filter out spatial background noise while refining and preserving robust local features.

The parallel Frequency Domain Branch (FDB) captures the global distribution characteristics of the image and the road context information, as differences specific to different domains, such as illumination variations and sensor styles, are often concentrated in specific frequency bands. For an input feature *F*, the branch first computes a two-dimensional real FFT and decomposes the complex spectrum into its amplitude |rFFT2(F)| and phase ∠rFFT2(F). Two 1×1 convolutions with BatchNorm/ReLU between them and a final sigmoid predict an amplitude mask *M* from the amplitude, and the branch reconstructs

(5) $$F_{freq}=\mathrm{irFFT2}\;\left(M\;\left(|\mathrm{rFFT2}(F)|\right)⊙\left|\mathrm{rFFT2}\left(F\right)\right|\;e^{\;j∠\mathrm{rFFT2}(F)}\right)$$

where ⊙ denotes element-wise multiplication, so that the learned gate modifies only the amplitude, preserves the original phase, and outputs a real-valued feature by the conjugate symmetry of the real-FFT representation; real and imaginary parts are never processed independently. The spatial and frequency outputs are summed, restoring the stage width *C*. As quantified in Section 3.5, the learned masks are depth-dependent amplitude reweightings rather than a uniform low-pass filter, so we do not claim that DIFA uniformly removes high-frequency noise.

In the feature fusion and adversarial discrimination stage, the fused features pass through a Gradient Reversal Layer (GRL) and are input into the domain discriminator. During the forward propagation of the network, the GRL keeps the features unchanged; however, during backpropagation, the GRL reverses the gradients, thereby inducing an adversarial game between the encoder and the discriminator. This forces the encoder to continuously optimize and extract domain-invariant features that render the discriminator unable to distinguish the source of the data.

Each training-only discriminator first projects the width-*C* stage feature to C/2 channels with a 1×1 convolution; its densely connected 3×3 blocks likewise operate at width C/2, followed by a 1×1 domain output layer. All concatenated branches are therefore of equal width C/2, so no channel-imbalance suppression of deep features arises. Spectral normalization is applied to all discriminator convolutions. The discriminator receives the full class-balanced domain classification signal, while the encoder receives only the reversed gradient whose coefficient is capped at 0.5 and scaled by $\lambda_{adv}$ (Section 2.4.2).

### 2.4. Loss Functions

To synergistically optimize the various functional modules of MAIG-Net and equip it with robust road extraction and generalization capabilities in complex cross-domain scenarios, this paper constructs a loss function system guided by multi-criteria joint constraints. Specifically, it first utilizes the complete label information of the source domain to establish a basic supervised segmentation constraint, endowing the model with preliminary road recognition capability; the same supervision is applied to the FDA intermediate images so that the network learns source road structure under target-like appearance. Secondly, it drives the DIFA modules through a multi-layer adversarial loss to achieve distribution alignment between the source and target domains in the feature space. Finally, it introduces a dual constraint mechanism based on intermediate domain guidance, which filters and routes detached teacher predictions into the discriminators at the pixel level and the topology-class level, respectively; this mechanism contributes no separate loss term but instead conditions the adversarial loss, so the overall objective is the weighted sum of the supervised and adversarial terms only (Section 2.4.3).

#### 2.4.1. Source Domain Supervised Segmentation Loss

The source domain supervised segmentation loss is the foundation for MAIG-Net to extract road features. Since road extraction from remote sensing imagery is essentially a pixel-level binary classification task, this paper adopts the Binary Cross-Entropy (BCE) loss as the basic supervision criterion:

(6) $$L_{BCE}=-\frac{1}{N}\sum_{i=1}^{N}\left[y_{i}\log\left({\hat{y}}_{i}\right)+\left(1-y_{i}\right)\log\left(1-{\hat{y}}_{i}\right)\right]$$

where *N* denotes the total number of pixels in a single image, $y_{i}$ represents the ground truth label of the *i*-th pixel, and ${\hat{y}}_{i}$ denotes the probability predicted by the network that the *i*-th pixel belongs to the road category. To strengthen region overlap and topological continuity of the elongated road class, the full supervised criterion combines BCE with Dice and soft centerline-Dice terms:

(7) $$L_{seg}\left(u,y\right)=0.5\;L_{BCE}\left(u,y\right)+0.5\;L_{Dice}\left(u,y\right)+0.1\;L_{clDice}\left(\sigma \left(u\right),y\right)$$

where *u* denotes the predicted logits and σ the sigmoid function. Let ρ(t) be zero through epoch 15 and then increase linearly with a 40-epoch denominator, capped at 0.4. With $w\left(t\right)=\lambda_{style}\;\rho \left(t\right)$ and $\lambda_{style}=1$, the supervised objective is the convex combination

(8) $$L_{sup}=\left(1-w\left(t\right)\right)\;L_{seg}\left(f\left(x_{s}\right),y_{s}\right)+w\left(t\right)\;L_{seg}\left(f\left(x_{st}\right),y_{s}\right)$$

where f(·) denotes the student segmentation network, *t* is the training epoch, and both terms use the source label $y_{s}$ only. This loss provides a stable semantic baseline for the entire network, ensuring that the model has acquired basic road recognition capabilities before undergoing any form of domain alignment, and serves as a crucial source of gradients supporting the subsequent multi-layer adversarial learning and dual constraint mechanism.

#### 2.4.2. Hierarchical Adversarial Alignment Loss

After establishing the basic road recognition capability, to bridge the distribution gap between the source and target domains, this paper introduces an adversarial learning mechanism utilizing the aforementioned DIFA modules. Because feature maps at different levels contain semantic information of various dimensions—ranging from shallow texture details to deep global topology—performing feature alignment solely at the end of the network can easily lead to the loss of local features. Therefore, this paper performs hierarchical adversarial alignment at three different stages of the encoder. For the set of aligned stages S={2,3,4} and stage discriminators $D_{l}$, the adversarial loss adopts the class-balanced binary domain cross-entropy

(9) $$L_{adv}=\frac{1}{2|S|}\sum_{l\in S}\left[\mathrm{BCE}\;\left(D_{l}\left(F_{s}^{l}\right),1\right)+\mathrm{BCE}\;\left(D_{l}\left(F_{t}^{l}\right),0\right)\right]$$

where $F_{s}^{l}$ and $F_{t}^{l}$ represent the features output by the *l*-th DIFA module for the source and target domains, respectively, and $D_{l}\left(\cdot \right)$ denotes the probability predicted by the *l*-th discriminator that the input feature originates from the source domain. For readability, Equation (9) suppresses the condition indices: each discriminator is evaluated per topology class, and target-side terms are weighted by the detached reliability *r* of Section 2.4.3, so unreliable target pixels contribute no alignment signal.

During the training process, facilitated by the synergistic effect of the GRL, a dynamic adversarial game is formed within the network. The domain discriminator strives to accurately distinguish the source of the features, while the encoder receives reversed gradients during backpropagation in an effort to deceive the discriminator. Through this mechanism, the loss forces the backbone network to generate domain-invariant features across multiple scales that render the discriminator unable to identify their origin. The discriminators receive the full domain classification signal; on the encoder side, the gradient-reversal coefficient is ramped and capped at 0.5 and the whole signal is scaled by $\lambda_{adv}=0.005$. Gradient norms are clipped at 1 for the main network and 10 for the discriminators.

#### 2.4.3. Dual Constraint Mechanism Based on Intermediate Domain Guidance

After achieving preliminary alignment in the feature space through the multi-layer adversarial modules, to further suppress the influence of unreliable target predictions under complex target-domain backgrounds, this paper introduces a dual constraint mechanism based on intermediate domain guidance. The two constraints act on which target pixels may condition the discriminators: a pixel-level reliability constraint and a topology-consistency constraint. Two alternative designs were deliberately rejected. A result-consistency loss on spatially stitched images is superseded by the FDA intermediate domain of Equations (3) and (4), which requires no target pseudo-label. A prototype-alignment loss that matches an intermediate prototype to the direct sum of source and target prototypes is mathematically ill-posed: the direct summation is neither a convex interpolation nor scale-invariant, so its distance to an “intermediate” prototype does not admit a well-defined interpretation. No target prediction is used as a direct segmentation label anywhere in MAIG-Net.

The source road mask and the teacher target prediction thresholded at 0.5 are each partitioned into five mutually exclusive topology classes: background, road interior, boundary, endpoint, and junction. Let $p_{T,i}$ and $p_{S,i}$ denote the teacher and student probabilities at target pixel *i*, and $z_{T,i}$, $z_{S,i}$ their topology classes. The target-pixel reliability is defined as

(10) $$r_{i}=\left(1-H(p_{T,i})\right)\;1\;\left[p_{T,i}\geq \tau \;\vee \;p_{T,i}\leq 1-\tau \right]\exp\;\left(-\frac{|p_{T,i}-p_{S,i}|}{T}\right)1\;\left[z_{T,i}=z_{S,i}\right]$$

where 1[·] is the indicator function, ∨ denotes logical disjunction, the binary entropy *H* is normalized to [0,1], the two-sided confidence gate uses τ=0.9, and the probability-stability temperature is T=0.15. Reliability values below 0.05 are set to zero, and the tensor *r* is detached from the computation graph before it conditions the discriminators. During the first 15 epochs, only source supervision is applied, so unstable early target predictions never participate in alignment.

In addition, a target-label-free foreground guard monitors the teacher road fraction as a global failure detector. The first 32 adaptation batches define a median baseline *b* of the predicted road fraction; thereafter, adaptation is suspended for a batch whenever the mean over the latest 16 batches exceeds the threshold

(11) $$q=\max\left(1.5\;b,\;b+0.03\right)$$

which detects persistent abnormal inflation of the predicted road area without reading any target mask. All quantities in this subsection are computed from predictions only. Section 3.5 quantifies the contribution of the reliability weighting and the topology conditioning under controlled perturbations.

#### 2.4.4. Overall Loss Function

To synergistically optimize the various modules in MAIG-Net and achieve smooth knowledge transfer from the source domain to the target domain, this paper combines the aforementioned loss functions through a weighted sum to construct an end-to-end overall objective function:

(12) $$L_{total}=L_{sup}+\lambda_{adv}L_{adv}$$

where $L_{sup}$ and $L_{adv}$ are the supervised and adversarial losses of Equations (8) and (9), with $\lambda_{style}=1$ inside $L_{sup}$ and the adversarial weight $\lambda_{adv}=0.005$; the discriminators minimize their own domain BCE. The released implementation additionally exposes optional path-consistency, prototype-bridge, path-order, direct target pseudo-label segmentation, and gradient-penalty objectives; all of their coefficients ($\lambda_{path}$, $\lambda_{bridge}$, $\lambda_{order}$, $\lambda_{tgt}$, $\lambda_{gp}$) are set to zero in MAIG-Net, and none of these terms participates in any reported experiment.

During the training process, to ensure the initial stability of model convergence, this paper employs a warm-up mechanism in conjunction with the aforementioned final weights. During the first 15 epochs, the model relies solely on source-domain supervised learning; afterwards, the FDA supervision weight w(t) and the adversarial ramp increase gradually as described above. This joint optimization mechanism, progressing from easy to difficult and from source domain supervision to cross-domain unsupervision, effectively prevents drastic gradient oscillations in the early stages and enhances the stability of adaptation.

### 2.5. Training and Inference Protocol

Training uses AdamW with an initial learning rate of $3\times 10^{-4}$ and weight decay 0.01, a cosine schedule with an 80-epoch horizon and minimum learning rate $10^{-5}$, and 30 actual training epochs. At adaptation onset (epoch 16), the learning rate is multiplied by 0.5 and the running statistics of the encoder BatchNorm layers are frozen. The reported model is always the epoch-30 EMA teacher evaluated at road probability threshold 0.5. At inference, the image-level FDA and all domain discriminators are removed, while the EMA encoder–decoder and the three DIFA feature transforms are retained; this distinction separates training-only appearance operations from the deployed model. Target labels do not enter training gradients, learning-rate or resumption decisions, checkpoint selection, or threshold selection.

## 3. Experimental Results and Analysis

### 3.1. Dataset Description

To comprehensively evaluate the feature transfer and generalization capabilities of the proposed MAIG-Net model in unsupervised domain adaptation tasks, this section constructs cross-domain road extraction experimental tasks with progressive levels of difficulty. The experiments involve three high-resolution remote sensing datasets, where SpaceNet serves as the source domain providing foundational labels, while DeepGlobe and Massachusetts act as the target domains to be adapted. A matched reverse direction, DeepGlobe→SpaceNet, is additionally evaluated.

#### 3.1.1. Source Domain Dataset (SpaceNet)

This dataset contains satellite imagery from several representative cities, including Las Vegas, Paris, Shanghai, and Khartoum, with a spatial resolution of approximately 0.3 m. The dataset is characterized by clear road topological structures, and its backgrounds are primarily dominated by highly structured urban buildings and suburbs. Given its accurate and rich label information, SpaceNet is highly suitable as the source domain data to provide foundational road semantic knowledge. In the cross-domain experiments of this paper, 2041 labeled images constitute the source training set, and an independent 254-image SpaceNet test set is reserved for the reverse direction.

#### 3.1.2. Target Domain Datasets (DeepGlobe and Massachusetts)

DeepGlobe Road Dataset: This dataset is a challenging collection of high-resolution satellite imagery constructed for the CVPR 2018 DeepGlobe Road Extraction Challenge [22]. It contains a total of 6226 images with dimensions of 1024×1024 pixels and a ground spatial resolution of 0.5 m. The captured locations of these images cover multiple countries such as Thailand, India, and Indonesia, encompassing a rich variety of geographical scenes including urban, suburban, and rural areas. In the forward protocol, the locally available 6226-image pool serves as the unlabeled target during training, and all images are evaluated only after training; no separate labeled DeepGlobe holdout is available, a limitation disclosed in Section 3.3. Massachusetts Road Dataset [23]: This dataset serves as another authoritative public benchmark, consisting of aerial imagery of the city of Boston and its surrounding areas. All images have uniform dimensions of 1500×1500 pixels at a nominal resolution of 1.0 m/pixel. The protocol uses 1103 unlabeled target training images, a 49-image test set, and a previously untouched 14-image validation holdout; both evaluation sets are disjoint from the target training set, so no holdout result reflects checkpoint selection on test data.

Under the unsupervised domain adaptation setting of this study, the core consideration for selecting them as the target domains lies in their ability to provide highly representative domain shift challenges. Specifically, the adaptation from SpaceNet to DeepGlobe evaluates the model’s transfer capability across different geographical regions and complex backgrounds. DeepGlobe contains a vast amount of unstructured natural backgrounds, such as forests and farmlands, along with severe tree occlusions. In this task, the model is required to demonstrate its anti-interference and feature decoupling capabilities under drastic background variations. Meanwhile, the adaptation from SpaceNet to Massachusetts evaluates the model’s capability to adapt low-level features across different sensors and resolutions. The Massachusetts dataset consists of aerial imagery, which exhibits significant physical-level discrepancies from the satellite imagery of SpaceNet in terms of imaging sensors, flight altitude, color saturation, and contrast. The matched reverse experiment uses the 6226 labeled DeepGlobe images as the source, the 2041 SpaceNet training images as the unlabeled target (153.6-m physical crops at 0.3 m/pixel output), and the independent 254-image SpaceNet test set for evaluation.

### 3.2. Experimental Details

All experiments are implemented in PyTorch 2.8.0 with Python 3.12, and training and evaluation are conducted on a single NVIDIA GeForce RTX 5090 GPU. During data loading, GSD-matched crops of 512×512 pixels (Section 2.1) are augmented with random horizontal and vertical flips. The default physical batch is 12 in FP32; configurations that exceed memory use a physical batch of 4 with three-step gradient accumulation to preserve the effective batch of 12. All primary runs use the 30-epoch schedule and the optimization settings of Section 2.5. Road-class IoU, F1-score, precision, and recall are computed from full-image confusion counts over each evaluation set. Every primary result is the fixed epoch-30 EMA teacher at threshold 0.5, reported as mean ± sample standard deviation (denominator n−1) over three seeds, with paired differences computed within the same seed. Because only three seeds are available, no significance claim is attached; per-seed paired differences are given instead. One aspect is disclosed explicitly: the final FDA-plus-adversarial branch combination was selected after inspecting seed-1 target metrics, so the additional seeds establish repeatability of a post-selected configuration rather than pre-registered independent confirmation.

### 3.3. Comparative Experimental Results

To intuitively demonstrate the performance of MAIG-Net, this paper comprehensively compares its segmentation results with those of various advanced domain adaptation methods. The baseline models selected for comparison not only encompass classic general domain adaptation methods based on self-training and uncertainty—such as CBST [24], CRST-MRKLD [25], Model-Uncertainty [26], and MLSL-SISC [27]—but also include models specifically designed in recent years for remote sensing road extraction, namely RoadDA [28], TGN [5], GOAL [5], and DOER [29]. The detailed quantitative comparison results are presented in Table 1. Table 1 separates two explicitly labeled evidence levels. The upper panel presents the baseline comparison as point estimates quoted from the respective publications, obtained under their originally reported protocols and evaluation environments. The lower panel reports the results of this study under the matched protocol defined in Section 3.2: GSD-aware sampling, a fixed 30-epoch budget with the epoch-30 EMA teacher, threshold 0.5, and three seeds reported as mean ± sample standard deviation. Because the two panels differ in data splits, training budgets, and checkpoint-selection rules, no ranking claim is made across panels; the direct like-for-like comparison of MAIG-Net is against its seed-matched source-only control in the same panel.

On SpaceNet→DeepGlobe, the three paired MAIG-Net-minus-source-only IoU differences are +0.06462, +0.04449, and +0.03783, giving a mean paired gain of +0.04898±0.01395; all three pairs are positive. This establishes repeatability across the recorded seeds but, per the disclosure in the previous subsection, not independent confirmation of the post-selected configuration, since no separate labeled DeepGlobe holdout is available for such a check. The adaptation increases mean recall by 0.0829 while reducing precision by 0.0466, so the gain reflects a recall–precision trade-off rather than a uniform improvement of all error types. On the 49-image Massachusetts test set, the paired IoU gain is +0.00824±0.00393. After freezing the method, checkpoint epoch, and threshold, the previously untouched 14-image validation holdout was evaluated once: MAIG-Net/source-only IoU is 0.4310±0.0016/0.4227±0.0028, a paired gain of +0.00826±0.00322. The consistent direction is encouraging, but the effect is small and shows the same precision-for-recall trade.

Iqbal et al. [7] report 0.462/0.632 and 0.340/0.507 on the same named directions; because their protocol differs in labeled-source size, crop geometry, batch size, pseudo-label rounds, and target split, these values are presented only as published-protocol context. De-GLGAN [13] and the disentangled representation of Lu et al. [14] receive no numerical cells, because no verifiable released result on these road directions was identifiable; quoting numbers from unrelated benchmarks, or rerunning methods without released road configurations, would not constitute a fair comparison.

**Figure 5:**
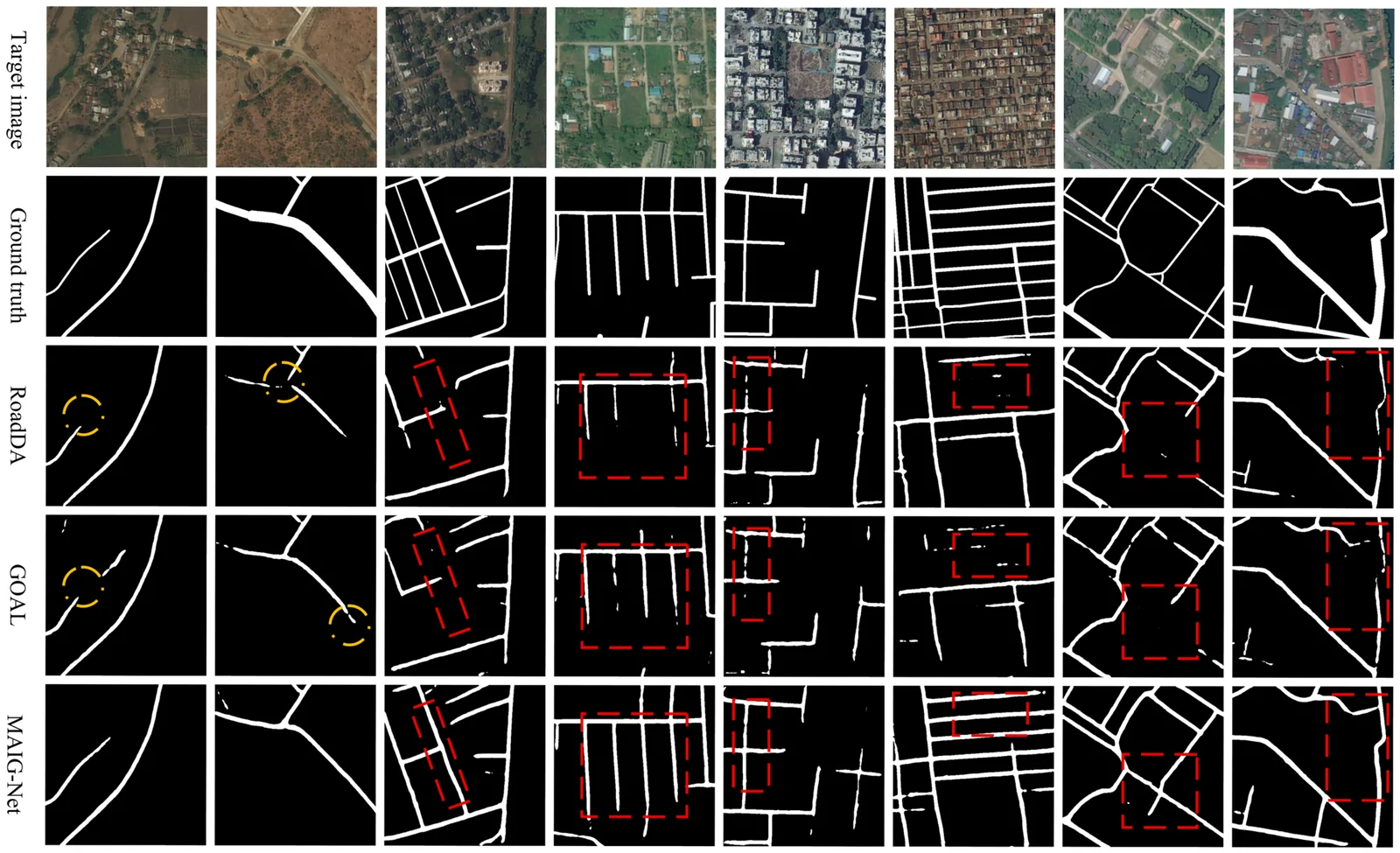
Visualization results on the DeepGlobe dataset.

To more intuitively demonstrate the behavior of the proposed method in cross-domain road extraction tasks, prediction results of several typical scenes were selected from the two target domain test sets for qualitative comparative analysis. Figure 5 illustrates the qualitative comparison for the SpaceNet to DeepGlobe task. In the figure, the first row displays the original target domain images, the second row shows the ground truth labels, and the subsequent rows present the extraction results of RoadDA [28], GOAL [5], and the proposed MAIG-Net, respectively. Red dashed boxes and yellow dashed circles highlight the key areas of discrepancy.

The DeepGlobe dataset contains a substantial number of complex non-urban background scenes, such as farmlands, wastelands, and areas with tree occlusions. Due to the drastic discrepancies in background distributions between the source and target domains, RoadDA exhibits severe missed detections in these complex scenes (as indicated by the red dashed boxes in columns 3, 4, and 5), making it almost impossible to extract a complete road network. Although the GOAL model mitigates the missed detections to a certain extent, it still suffers from road edge blurring and topological disconnections when encountering tree occlusions or low-contrast road segments (as indicated by the yellow dashed circles in columns 1 and 2). In these selected scenes, MAIG-Net extracts more complete road networks with clearer boundaries, and maintains geometric connectivity at the curves and intersections in columns 1 and 2. These examples are illustrative rather than statistically representative; the failure cases in Section 3.6 show scenes in which MAIG-Net also fails completely.

**Figure 6:**
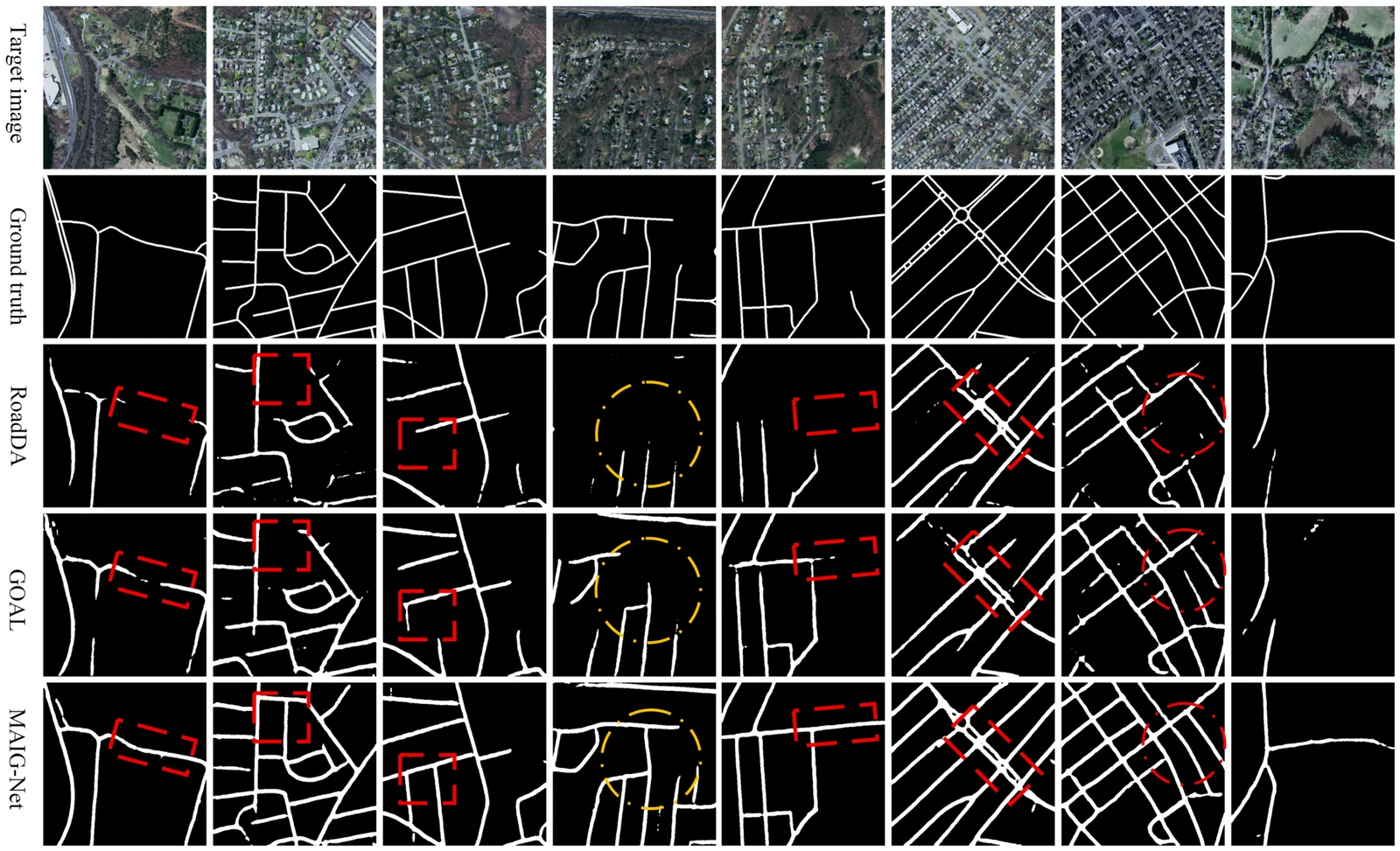
Visualization results on the Massachusetts dataset. The red dashed boxes in columns 3, 5 and 6 show that our method achieves favorable results with well-preserved road-extraction connectivity.

Figure 6 illustrates the qualitative comparison for the SpaceNet to Massachusetts task. In the area marked by the yellow dashed circle in column 4, RoadDA loses the features of this road segment, and GOAL only extracts scattered, fragmented pixels; in the complex multi-branch intersection areas highlighted by the red dashed circles in columns 7, the compared methods exhibit structural disconnections and morphological distortions. In contrast, in areas covered by dark shadows or with similar backgrounds (the red boxes in columns 1 and 2), MAIG-Net more often separates the road foreground from the heterogeneous background, and at the complex intersections in columns 6 and 7 it connects the truncated narrow road segments while preserving structural details. Consistent with the quantitative results, these qualitative differences indicate improved recall of road structure under appearance shift, obtained at some cost in precision.

### 3.4. Ablation Studies and Sensitivity Analysis

To investigate the specific contributions of each component of MAIG-Net, detailed diagnostic experiments are conducted on the GSD-matched SpaceNet→DeepGlobe task. All rows of Table 2 use seed 1, the fixed epoch-30 EMA teacher, and threshold 0.5, with one factor varied at a time and all other settings held fixed; because these diagnostics were run after the operating point was chosen, they are descriptive post-selection evidence rather than independent statistical confirmation of each choice.

Component and coefficient analysis. FDA and adversarial alignment together outperform either component alone, indicating that appearance interpolation and feature alignment address complementary parts of the shift. Along the $\lambda_{adv}$ axis, the selected value 0.005 is highest in the sampled sequence, but the curve is not a wide plateau; along the $\lambda_{style}$ axis, the best value lies at the sampled endpoint, so no claim of an interior optimum is made. F1 and IoU rank the settings identically, which excludes the possibility that the operating point appears favorable under only one overlap metric. Across the sampled ranges, stronger adaptation consistently raises recall and lowers precision.

Reliability and conditioning. Relative to the final setting, removing the pixel reliability reduces IoU by 0.02471 and flipping 10% of the topology-condition classes reduces it by 0.02488, and both variants mainly lower recall. These perturbations act on the discriminator conditioning that the final method actually uses—there is no direct pseudo-label segmentation loss to perturb—and support the contribution of the filtering and routing mechanisms in this setting, without establishing that τ=0.9 is optimal for other target domains. None of these runs triggered the foreground guard, automatic retries, or non-finite losses.

EMA, alignment depth, and architecture. Fixed EMA momentum 0.999 exceeds fixed 0.99 and the tested linear schedule; with only 15 adaptation epochs after warm-up, a schedule starting at 0.95 weights early target predictions more heavily without measured benefit. Three-stage alignment is 0.02299 IoU above stage 4 alone and 0.02266 above stages 3/4; since no stage-2-only run was performed, this is interpreted as a benefit of the three-depth combination rather than an isolated causal contribution of stage 2. The bottleneck spatial branch exceeds CBAM by 0.00685 IoU while CBAM uses 10.50% fewer parameters and 7.55% fewer counted FLOPs yet is 0.23 ms slower in the internally matched timing, showing that parameter count alone does not determine latency; the plain full-width branch is clearly weaker despite more parameters. A non-local variant is not reported because its quadratic spatial attention does not admit a memory-matched substitution at multiple 512-pixel stages under this protocol. The paired MAIG-Net-minus-source-only difference is positive for all three tested backbones, though the ResNet-18 gain is small, and the ResNet-50 source-only control triggered one pre-configured source-loss guard at epoch 16, batch 56 and completed after resuming at half learning rate; extending conclusions to transformer backbones would require separate controlled experiments.

Computational cost. Table 3 reports the deployment cost of MAIG-Net measured on the RTX 5090 with FP32, batch 1, 512×512 inputs, 5 warm-up and 100 synchronized iterations. The deployed graph contains the backbone, the decoder, and all three DIFA branches; the image-level FDA and the domain discriminators (4.05 M parameters) are training-only and never enter deployment. Relative to bypassing DIFA, the full model adds 14.31% parameters, 9.01% counted operator FLOPs, and 37.89% latency. The operator counter does not include FFT kernels, so an analytic 0.050 G estimate for the three RFFT/IFFT pairs is reported separately rather than folded into the counted total. The 4.80 ms figure comes from the standalone deployment profiling; an internally matched variant-timing batch measured the same configuration at 4.66 ms (Table 2), and the 0.14 ms discrepancy is treated as run-to-run timing variation across timing batches, not an architectural difference. Regarding memory, batch-12 FP32 training reaches 18.74 GiB allocated (19.58 GiB reserved) during adaptation, versus 6.02 GiB allocated during the source-only warm-up, indicating that the target/discriminator paths dominate training memory; 16 GiB GPUs therefore require a smaller physical batch with gradient accumulation to preserve the effective batch of 12.

### 3.5. Reliability and Frequency Analyses

Beyond the reliability rows of Table 2, the frequency behavior of the method is quantified directly. Across 64 target-label-free, GSD-matched source–target pairs (analysis seed 20260729, training-maximum FDA strength λ=0.45, frozen seed-1 checkpoint), the centered β=0.05 FDA window reduces the within-window mean log-amplitude distance between domains from 0.4791 to 0.3004 (a 37.29% reduction), while the full-spectrum distance changes by only 1.61%—consistent with the design in which coefficients outside the window are untouched. The small full-spectrum change should not be misread as a weak within-window operation; the two statistics answer different questions (intended effect versus locality check).

The learned DIFA masks have mean weights 0.4664, 0.5342, and 0.4526 at stages 2, 3, and 4, with clearly different radial responses: low/mid/high-frequency radial means are 0.5699/0.4773/0.4440 at stage 2, 0.5373/0.5322/0.5347 at stage 3, and 0.3705/0.4434/0.4708 at stage 4, with 5th–95th percentile ranges of 0.0687–0.8174, 0.1115–0.8933, and 0.1355–0.6055, respectively. Stage 2 thus emphasizes low frequencies, stage 3 is approximately flat, and stage 4 emphasizes high frequencies (Figure 3). This supports describing DIFA as phase-preserving, depth-dependent amplitude reweighting rather than uniform high-frequency removal, and the visualization is used to confirm model behavior only; segmentation effects are attributed through the matched-protocol ablations, not through the mask images.

### 3.6. Reverse Adaptation and Failure Cases

Reverse direction. In matched DeepGlobe→SpaceNet adaptation, MAIG-Net/source-only IoU is 0.24537±0.01289/0.23350±0.00341 and F1 is 0.39393±0.01673/0.37858±0.00449. The per-seed paired IoU differences are −0.00271, +0.02270, and +0.01563 (mean +0.01187±0.01311). Because only two of three pairs improve and the mean change comes mainly from recall, no claim of stable reverse improvement or bidirectional robustness is made. All six run directories contain completion markers, run configurations, metrics, final teacher, and resumption checkpoints, with no guard, retry, traceback, or non-finite event in the logs.

Failure cases. Using the three final checkpoints and their seed-matched source-only controls, all 6226 DeepGlobe target images were analyzed. All strata were defined by deterministic rules before any qualitative example was inspected: background similarity combines background RGB means/standard deviations with the normalized radial spectrum of local high-pass residuals, scored by each target image’s distance to its nearest source sample; the remaining decile proxies use the dark-background fraction, the green-excess background fraction, the overlap of dark or green regions with labeled roads, and the road-pixel density. These are reproducible stress proxies rather than subjective scene labels. Table 4 reports the mean IoU in each highest-stress decile (623 images each).

**Figure 7:**
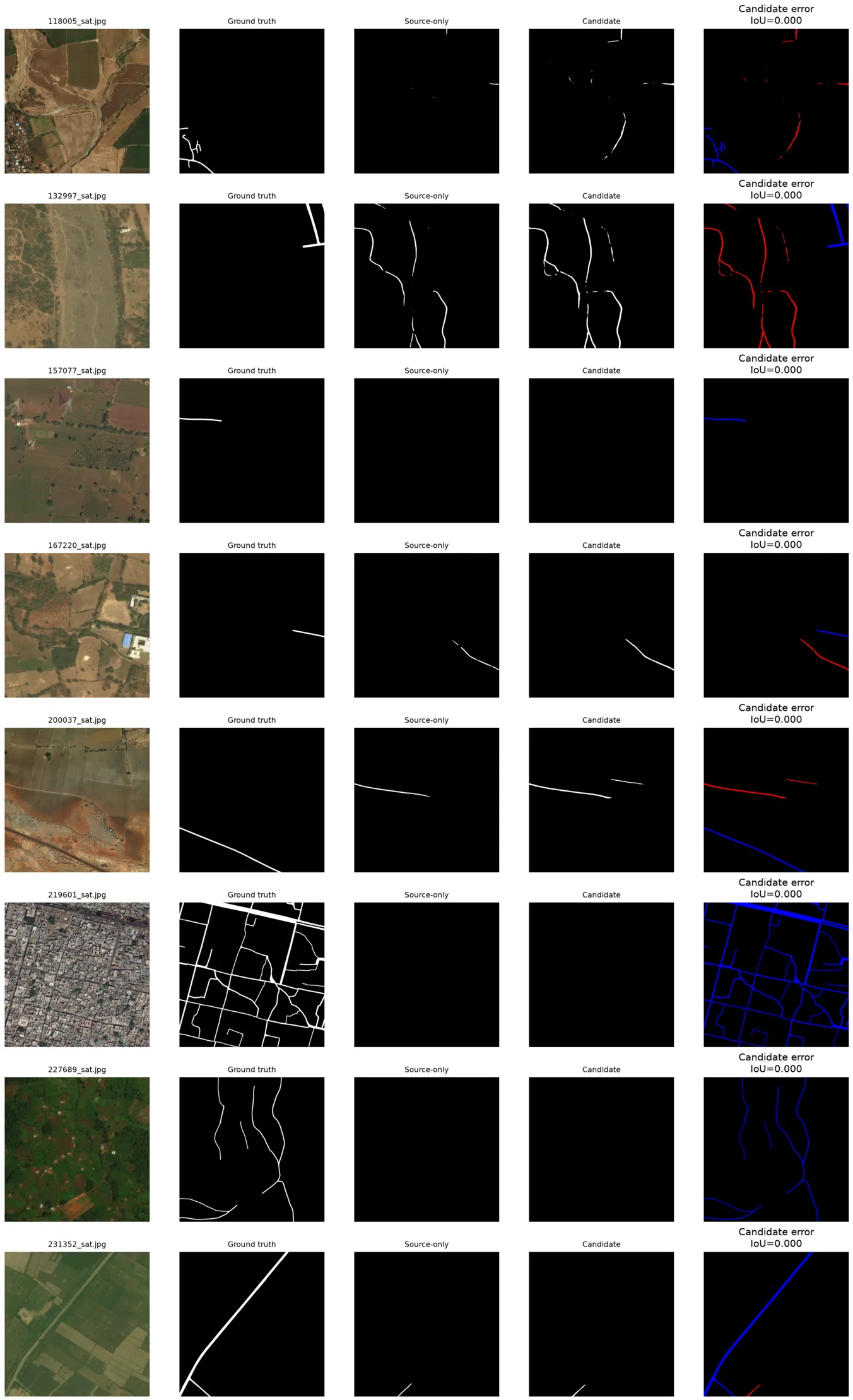
Eight lowest-IoU road-containing DeepGlobe tiles for the frozen seed-1 model. Columns show the input, ground truth, matched source-only prediction, MAIG-Net prediction, and MAIG-Net error map (red: false positive; blue: false negative).

All five strata retain a positive mean paired difference, so the data do not support the hypothesis that source-similar unstructured backgrounds specifically reverse the adaptation gain. However, the background-similarity and dense-road strata fall below the full-set MAIG-Net IoU of 0.3763, every stratum shows the precision-down/recall-up trade, and in the eight lowest-IoU road-containing tiles the MAIG-Net IoU is exactly zero (source-only is also zero in seven). These tiles include sparse farmland and dirt roads, low-contrast vegetated roads, and dense urban grids, with complete omissions and occasional linear false positives (Figure 7). These cases bound the applicability of the method and contradict any claim that frequency processing eliminates complex-background failures; the per-image metrics and proxy definitions are provided in the supplementary analysis for independent recomputation.

## 4. Conclusions

To address the critical challenges of model performance degradation caused by cross-domain data distribution discrepancies and the scarcity of annotated target domain data in high-resolution remote sensing road extraction, this paper proposes an unsupervised domain adaptation road extraction method based on multi-layer adversarial learning and intermediate domain guidance, termed MAIG-Net. The method controls physical scale before adapting appearance and features: GSD-aware sampling matches the source and target fields of view; a whole-image FDA intermediate domain provides source-labeled low-frequency appearance variation; and DIFA modules perform reliability-weighted, topology-conditioned adversarial alignment at three encoder depths. The EMA teacher prediction is detached and used only to condition the discriminators, so the method contains no direct target pseudo-label segmentation objective. Under the matched protocol, all three SpaceNet→DeepGlobe seed pairs improve over source-only training, and SpaceNet→Massachusetts shows a smaller, directionally consistent gain on both the test set and a previously untouched validation holdout. These findings are bounded by the disclosed post-selection on DeepGlobe, a consistent precision–recall trade-off, complete failure on several hard road-containing tiles, and seed-dependent reverse adaptation. The evidence therefore supports MAIG-Net as a reproducible advance over source-only training under matched physical scale, rather than a claim of universal superiority or stable bidirectional transfer. Future work will focus on exploring the potential of this method in multi-source heterogeneous remote sensing data fusion and lightweight model deployment.

## Figures and Tables

**Table 1:** Quantitative comparison results of cross-domain road extraction. “Published results” are point estimates quoted from the respective papers; “This study” rows follow the protocol of Section 3.2 and report mean ± standard deviation over three seeds. Values are not directly rankable across the two panels.

Model	SpaceNet→DeepGlobe		SpaceNet→Massachusetts	
	IoU	F1	IoU	F1
Published results				
DOER [29]	0.328	0.494	0.289	0.435
RoadDA [28]	0.233	0.363	0.205	0.319
TGN [5]	0.278	0.436	0.245	0.384
MLSL-SISC [27]	0.396	0.565	0.301	0.461
CRST-MRKLD [25]	0.330	0.496	0.295	0.456
Model-Uncertainty [26]	0.334	0.501	0.298	0.460
CBST [24]	0.390	0.561	0.284	0.443
GOAL [5]	0.358	0.527	0.351	0.520
Iqbal et al. (2023) [7]	0.462	0.632	0.340	0.507
This study				
GSD-matched source-only	0.3274±0.0027	0.4932±0.0031	0.4498±0.0032	0.6205±0.0031
MAIG-Net (ours)	0.3763±0.0126	0.5468±0.0132	0.4580±0.0007	0.6283±0.0007

**Table 2:** Post-selection, seed-1 diagnostic results on GSD-matched SpaceNet→DeepGlobe (fixed epoch-30 EMA teacher, threshold 0.5). One factor is varied at a time with all other settings held fixed. In the backbone panel, accuracy entries are MAIG-Net/source-only under identical seeds and budgets. The plain spatial branch and both ResNet-50 runs used a physical batch of 4 with three-step gradient accumulation.

Axis	Setting	IoU	F1	Note
Adaptation	Source-only	0.32623	0.49197	$\lambda_{style}=\lambda_{adv}=0$
	FDA only	0.34901	0.51743	$\lambda_{style}=1$, $\lambda_{adv}=0$
	Adversarial only	0.32958	0.49576	$\lambda_{style}=0$, $\lambda_{adv}=0.005$
	FDA + adversarial	0.39085	0.56203	Final configuration
$\lambda_{adv}$ ($\lambda_{style}=1$)	0	0.34901	0.51743	
	0.0025	0.37066	0.54085	
	0.005	0.39085	0.56203	Final configuration
	0.01	0.37357	0.54394	
$\lambda_{style}$ ($\lambda_{adv}=0.005$)	0	0.32958	0.49576	
	0.5	0.36685	0.53678	
	1	0.39085	0.56203	Final; sampled endpoint
Reliability	τ=0.8	0.37239	0.54269	
	τ=0.9	0.39085	0.56203	Final configuration
	τ=0.95	0.37547	0.54595	
	No pixel reliability	0.36614	0.53602	Foreground guard kept
	10% topology flip	0.36597	0.53583	Conditioning perturbed
EMA	Fixed 0.99	0.36060	0.53005	
	Linear 0.95→0.999	0.36102	0.53051	
	Fixed 0.999	0.39085	0.56203	Final configuration
Alignment stages	Stage 4	0.36786	0.53786	
	Stages 3/4	0.36819	0.53822	
	Stages 2/3/4	0.39085	0.56203	Final configuration
Spatial branch	Plain	0.33415	0.50092	41.90 M, 66.36 G, 4.65 ms
	CBAM	0.38400	0.55491	32.65 M, 55.49 G, 4.89 ms
	Bottleneck	0.39085	0.56203	36.48 M, 60.02 G, 4.66 ms
Backbone	ResNet-18	0.34524/0.33379	0.51328/0.50051	Paired IoU +0.01145
	ResNet-34	0.39085/0.32623	0.56203/0.49197	Paired IoU +0.06462
	ResNet-50	0.35336/0.29608	0.52220/0.45689	Paired IoU +0.05728

**Table 3:** Deployment cost on the RTX 5090 (FP32, batch 1, 512×512 input; 5 warm-up and 100 synchronized iterations). Counted FLOPs exclude FFT kernels, whose analytic estimate is 0.050 G for the three RFFT/IFFT pairs. The training-only FDA and domain discriminators are excluded from both rows.

Deployed Path	Params (M)	Counted FLOPs (G)	Latency (ms)	FPS
Backbone and decoder, DIFA bypassed	31.91	55.05	3.48	287.4
MAIG-Net (with DIFA)	36.48	60.02	4.80	208.4
Increment	+14.31%	+9.01%	+37.89%	—

**Table 4:** Stress-stratified mean road IoU over the full 6226-image DeepGlobe pool (three final checkpoints and seed-matched source-only controls). Each stratum is the highest decile of a deterministic proxy defined before qualitative inspection.

Stress Stratum	MAIG-Net IoU	Source-Only IoU	Paired Difference
Highest background similarity	0.3549	0.3026	+0.0523
Shadow-like decile	0.3582	0.3034	+0.0547
High vegetation decile	0.4491	0.4055	+0.0436
Canopy-like road overlap decile	0.4347	0.3936	+0.0411
Dense road network decile	0.3337	0.2695	+0.0642

## Data Availability

Supporting code is compiled and accessible via the following GitHub link: https://github.com/xiaozaizaidezaizai/Road-domain-adaptation (accessed on 24 April 2026). The SpaceNet [30], DeepGlobe [22], and Massachusetts Roads [23] datasets are publicly available from their original providers.
